# Innovations in collagen-network remodeling and extracellular matrix mechanics: toward a new era in articular cartilage repair

**DOI:** 10.3389/fbioe.2025.1740135

**Published:** 2026-01-06

**Authors:** Kai Huang, Yifan Hong, Haili Cai

**Affiliations:** 1 Tongde Hospital of Zhejiang Province, Hangzhou, China; 2 Zhejiang Chinese Medical University, Hangzhou, China; 3 The 903rd Hospital of the People’s Liberation Army, Hangzhou, China

**Keywords:** articular cartilage, collagen fiber organization, extracellular matrix dynamics, mesenchymal stem cells, osteoarthritis, viscoelastic properties

## Abstract

Articular cartilage is a highly specialized connective tissue with a hierarchically organized extracellular matrix (ECM) that provides the mechanical resilience necessary for joint function. Central to this functionality is the depth-dependent architecture of collagen—primarily type II—interwoven with proteoglycans, enabling efficient resistance to compressive and shear stresses. This review synthesizes recent advances in ECM dynamics, emphasizing the interplay between collagen organization, viscoelastic microenvironments, and pericellular-matrix (PCM)–mediated mechanotransduction. Emerging evidence implicates type III collagen as a regulator of early cartilage remodeling and a putative biomarker of osteoarthritis (OA) progression. Additionally, we highlight cutting-edge studies on the synergistic effects of mechanical loading and enzymatic degradation on collagen integrity, providing novel insights into ECM deterioration in disease contexts. We evaluate next-generation biomaterials—including viscoelastic hydrogels, anisotropic scaffolds, and magnetic field–assisted fiber alignment—designed to recapitulate the native anisotropy and multiscale mechanics of cartilage. Together, these recent developments redefine the landscape of cartilage repair and delineate promising avenues for translational regenerative therapies.

## Introduction

Articular cartilage is a specialized tissue whose ECM—composed predominantly of type II collagen and proteoglycans—underpins its durability and low-friction articulation under substantial compressive and shear loads. The collagen–proteoglycan network is essential for load bearing and low friction during motion (Pueyo et al., 2025). Depth-dependent (zonal) organization further modulates mechanics: stiffness generally increases with depth, and the tissue exhibits compression-induced strain-softening, features integral to function (Luo et al., 2017). Understanding how ECM structure dynamically interacts with chondrocytes is critical for repair strategies. Mechanical behavior derives not only from composition but also from hierarchical architecture, where depth-dependent fibril alignment couples with a proteoglycan-rich interstitial fluid to govern load response (Gottardi et al., 2016). Within this hierarchy, the pericellular matrix (PCM) mediates mechano-signaling, enabling bidirectional reciprocity between extracellular cues and cell responses—an essential feature of cartilage homeostasis (Gilbert et al., 2021). Despite its exceptional mechanics, articular cartilage is intrinsically avascular and exhibits limited self-repair, posing a persistent challenge for regenerative medicine. Current repair strategies prioritize recapitulating zonal structure and mechanics using advanced biomaterials and tissue-engineering approaches (Baei et al., 2023). Yet achieving native friction and wear remains difficult, constraining long-term construct durability (Joukar et al., 2023). This review explores the significance of ECM dynamics in cartilage function, repair, and regeneration.

## ECM dynamics in articular cartilage

**FIGURE 1:**
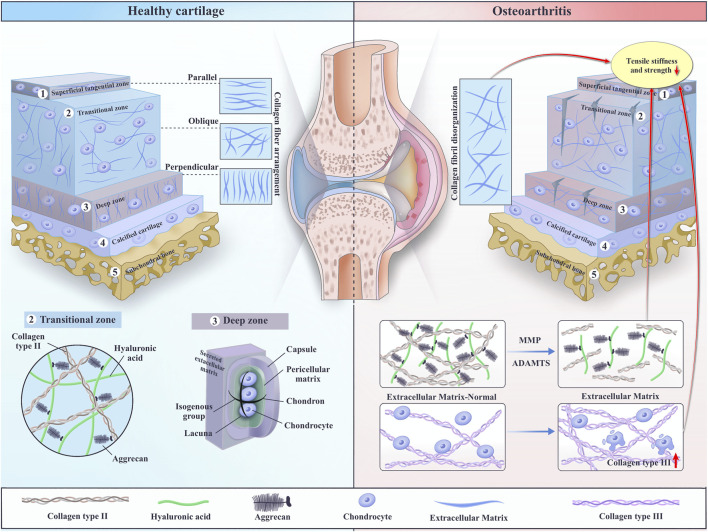
Comparative structure of healthy cartilage and osteoarthritic cartilage. Healthy cartilage is organized into the superficial tangential zone (STZ), transitional zone, deep zone, calcified cartilage, and subchondral bone, with collagen type II fibrils arranged in parallel, oblique, and perpendicular orientations depending on depth. In osteoarthritic cartilage, this orderly architecture is disrupted: the type II collagen network becomes fragmented and disorganized and there is increased deposition of type III collagen as part of extracellular matrix (ECM) remodeling, particularly at the calcified cartilage–subchondral bone interface. These compositional and architectural changes give rise to specific biomechanical deficits, including reduced tensile stiffness and strength in the superficial zone and impaired load-bearing capacity. Key ECM components such as proteoglycans (aggrecan), collagen type II, and hyaluronic acid are also highlighted, together with matrix-degrading enzymes such as MMPs and ADAMTS that drive progressive loss of cartilage structure and mechanics during osteoarthritis.

The ECM in articular cartilage plays a pivotal role in maintaining tissue homeostasis and regulating cellular responses. The composition and organization of the ECM vary across different zones of cartilage, with each zone performing distinct mechanical functions (e.g., compressive resistance in the deep zone and shear resistance in the superficial zone) (Table 1). The PCM, surrounding chondrocytes, is particularly important in modulating cell behavior through mechanotransduction (Figure 1).

**TABLE 1:** Native cartilage ECM: depth-dependent features, collagen composition, and assessment modalities.

Category	Item	Key attributes	Mechanical/Function	Limitations/Pathology	Assessment/Readouts	Refs
Zone	Superficial tangential zone (STZ)	Parallel to surface; high fibril density; low–moderate PG; high water; low modulus	Shear resistance; lubrication; load distribution	—	—	(Luo et al., 2017), (Hosseini et al., 2014), (Halonen et al., 2013), (McLeod et al., 2013)
	Transitional (middle) zone	Oblique/random fibrils; moderate PG & water; moderate modulus	Energy dissipation; stress transition	—	—	(Luo et al., 2017), (McLeod et al., 2013)
	Deep (radial) zone	Perpendicular/arcade; high PG; lower water; high modulus	Compressive load bearing; fluid pressurization	—	—	(Luo et al., 2017), (Halonen et al., 2013), (McLeod et al., 2013)
	Calcified cartilage	Mineralized interface; very low PG; lowest water; very high stiffness	Anchorage to bone; stress transfer	Early damage localization	—	(Hughes et al., 2021)
Collagen	Type II	Major fibrillar collagen; present across zones	Tensile and shear resistance; fibril architecture	Loss/disorganization in OA	—	(Pueyo et al., 2025), (Hosseini et al., 2014), (Halonen et al., 2013)
	Type III	Low in healthy; ↑ during remodeling	Regulates fibrillogenesis; fine fibrils	Elevated in OA; biomarker of remodeling	—	(Hosseininia et al., 2016), (Wang et al., 2020)
	Type X	Minimal in hyaline cartilage; hypertrophic regions	Hypertrophy/mineralization marker	Lower expression desirable in repair constructs	—	van Kampen et al. (2023)
Assessment	MRI T2 mapping	T2 relaxation reflects organization and water (indirect)	Sensitive to early organizational/water changes	Not specific to collagen concentration	T2 mapping	Lavalle et al. (2025)
	Musculoskeletal ultrasound	Backscatter/anisotropy; texture	Detects early collagen network changes	Operator-dependent; depth limits	Echotexture/anisotropy	Zhang et al. (2024)
	AFM micromechanics (research)	Indentation modulus; anisotropy	Resolves zonal/PCM stiffness; micromechanics	*Ex vivo*/research only	Indentation curves	McLeod et al. (2013)
	Split-line mapping/PLM (research)	Orientation patterns	Gold standard for fiber direction	*Ex vivo*; not clinical	PLM orientation maps	(Speer and Dahners, 1979), (Jeffery et al., 1991)

Abbreviations: STZ, superficial tangential zone; ECM, extracellular matrix; GAG, glycosaminoglycan; PCM, pericellular matrix; PLM, polarized light microscopy; AFM, atomic force microscopy.

### Collagen in cartilage ECM

Type II collagen is the principal fibrillar constituent of articular cartilage, while other collagens—most notably type III—support extracellular matrix (ECM) homeostasis. The depth-dependent, non-uniform distribution of collagen fibrils is critical for load sharing and underlies the tissue’s distinctive mechanical behavior. The superficial tangential zone (STZ), characterized by densely packed fibrils aligned parallel to the surface, is pivotal for lateral load redistribution (Figure 1). By shunting compressive loads from focal contact regions to adjacent areas, the STZ enhances overall load-bearing capacity. When compromised, cartilage exhibits altered mechanics—including increased stiffness and distinct deformation patterns—underscoring the STZ’s central role in maintaining function (Hosseini et al., 2014). Moreover, depth-wise distributions of collagen and proteoglycans govern the tissue’s response under dynamic joint loading (e.g., gait) and at mechanical equilibrium (e.g., quiet standing). Three-dimensional finite-element analyses of the human knee indicate that an arcade-like fibrillar architecture markedly reduces stresses during dynamic loading relative to homogeneous models. These depth-dependent variations in collagen and proteoglycan content shape local deformations and stress fields, highlighting the need to preserve native zonal organization in engineered constructs. Practically, a constant fibril volume density can be assumed in some 3D models, but realistic depth-wise proteoglycan gradients are essential for accurately capturing equilibrium responses (Halonen et al., 2013).

### Viscoelastic properties

The viscoelastic properties of the ECM—most prominently within the PCM—govern cell behavior by delivering mechanical cues. During cartilage regeneration, ECM viscoelasticity critically modulates chondrocyte functions, including matrix synthesis and lineage-specific differentiation. Ryu et al. developed enzyme-mediated crosslinking gelatin-based hydrogels that mimic the ECM’s mechanical properties. These hydrogels demonstrated enhanced adhesion to surrounding tissues, suggesting that the mechanical characteristics of the ECM can be effectively replicated to influence cell behavior and promote tissue regeneration (Ryu et al., 2022). This aligns with findings from other research indicating that biofunctionalized hydrogels can regulate cell behavior by mimicking biochemical cues present in the ECM (Cao et al., 2021). The mechanical microenvironment, including matrix stiffness and viscoelasticity, has been shown to significantly affect the behavior of various cell types, including chondrocytes (Zhang et al., 2016). Moreover, the transfer of stress and strain signals between the ECM and cells is vital for translating mechanical cues into biochemical signals. The PCM, which is a specialized region of the ECM surrounding cells, is particularly influential in this process. It has been noted that components like perlecan (HSPG2) within the PCM interact with cells to facilitate mechanosensing, thereby influencing cellular responses to mechanical stimuli (Boos et al., 2022).

## Collagen fiber organization and mechanical properties

The alignment and organization of collagen fibers significantly influence the mechanical properties of cartilage. Articular cartilage exhibits a layered structure, with collagen fibers organized into distinct zones: the superficial, transitional, and deep zones. This organization is essential for cartilage to function under compressive and shear loads.

### Depth-dependent collagen organization

The transition of collagen fiber orientation from parallel at the surface to perpendicular in the deep zone imparts anisotropic properties to the tissue, enabling cartilage to efficiently resist diverse mechanical stresses (Figure 1). Speer et al. provided early insights into the collagenous architecture of articular cartilage using scanning electron microscopy and polarized light microscopy, revealing the complex organization of collagen fibers. Their work established the foundation for understanding how collagen orientation influences cartilage’s mechanical properties (Speer and Dahners, 1979). Jeffery and his team further expanded this understanding by examining the three-dimensional collagen architecture in bovine articular cartilage. They observed that collagen in the superficial and intermediate zones forms leaf-like layers and fine meshwork, with anisotropic alignment across zones. Split-line patterns extending through the cartilage layers emphasized the importance of collagen fiber orientation in maintaining cartilage’s structural integrity and mechanical function under varying loading conditions (Jeffery et al., 1991). McLeod et al. employed atomic force microscopy to investigate the depth-dependent anisotropy of the ECM and PCM in articular cartilage. They found that ECM stiffness varies with depth, showing distinct anisotropy in the superficial and deep zones, while the middle zone exhibited subtle anisotropic behavior. ECM properties decreased with depth in all directions, while the PCM displayed uniform stiffness across zones, with higher moduli parallel to the split-line direction. These findings highlighted the complexity of cartilage’s micromechanical environment and its depth- and direction-dependent behavior (McLeod et al., 2013). Building on this, Kampen et al. introduced a hypotrochoidal scaffold design for cartilage tissue engineering. Under dynamic culture conditions, this design promoted increased collagen type II deposition, reduced collagen type X expression, and enhanced glycosaminoglycan synthesis in areas subjected to higher stress. These results reinforce the notion that collagen fiber orientation is crucial for the biomechanical properties of articular cartilage (van Kampen et al., 2023).

### Collagen III and fiber development

Type III collagen is increasingly recognized for its pivotal role in collagen fibrillogenesis, particularly during the early stages of cartilage development and repair (Figure 1). Recent studies have highlighted its significance in various contexts, including OA and the structural integrity of articular cartilage. Hosseininia et al. investigated the deposition of type III collagen in articular cartilage, suggesting that its presence may serve as a distinctive biomarker for hip OA. Their findings indicated that the amount of newly synthesized type III collagen was significantly higher in OA cartilage compared to reference cartilage, implying an active remodeling process in response to injury. This suggests that type III collagen not only contributes to the structural framework of cartilage but also plays a role in the pathological changes associated with OA (Hosseininia et al., 2016). Further supporting the importance of type III collagen, a study emphasized its crucial structural role in both articular cartilage and meniscus extracellular matrices. This research underscores the necessity of type III collagen in maintaining the integrity and functionality of cartilage, particularly during the early phases of development and repair. The regulation of collagen fibrillogenesis, which is essential for the formation of a stable collagen network, is significantly influenced by type III collagen, as it interacts with other matrix components to facilitate proper fibril assembly (Wang et al., 2020). In summary, type III collagen is a key regulator of collagen fibrillogenesis, particularly in the context of cartilage development and repair. Its increased deposition in osteoarthritic cartilage highlights its potential as a biomarker for disease progression and underscores its role in the active remodeling processes that occur in response to injury.

## Impact of pathological conditions on ECM and collagen

Joint injuries and diseases such as OA lead to changes in the ECM, which in turn affect cartilage’s ability to function and repair. These conditions result in the degradation of key ECM components, including collagen.

### Collagen loss and degradation in OA

Collagen is a critical structural protein that imparts strength and elasticity to cartilage. Loss of collagen integrity weakens cartilage, accelerating OA progression and causing joint pain and stiffness. A study using an explant model of early-stage post-traumatic osteoarthritis (PTOA) found that injurious loading resulted in a significant reduction of collagen near cartilage lesions immediately after injury, with further degradation observed by day 12. Notably, physiological cyclic loading was shown to partially preserve collagen in unaffected regions after 12 days, suggesting a potential therapeutic strategy for maintaining cartilage integrity in OA (Hamada et al., 2025). Microscopic analysis also reveals that cartilage injury disrupts the collagen matrix early on, leading to structural disorganization. OA-induced damage to the collagen network triggers chondrocyte proliferation and phenotypic changes, which are indicative of a response to matrix degradation (Sen and Hurley, 2025). Additionally, imaging techniques such as T2 relaxation times indicate changes in collagen organization and water content, though not directly linked to collagen concentration (Lavalle et al., 2025). Ultrasound have been utilized to detect early changes in collagen network organization. Abnormal collagen organization and composition are detectable in the early stages of cartilage injury, emphasizing the importance of collagen integrity in disease progression (Zhang et al., 2024). Overall, collagen degradation, particularly around cartilage lesions, is a key factor in OA development, contributing to the deterioration of cartilage mechanical properties and overall tissue integrity (Figure 1).

### Mechanical loading and collagen damage

The relationship between mechanical loading and collagen damage in cartilage has been widely investigated through experimental and computational methods. Hughes et al. highlighted that the calcified cartilage, susceptible to matrix damage likely involving collagen, may be the initial tissue affected by physiological loading and aging, especially in the context of ochronosis (Hughes et al., 2021). This suggests that mechanical stress plays a role in early collagen matrix alterations at osteochondral interfaces. Structural integrity and mechanical performance of cartilage are also influenced by its collagen architecture. Moo et al. demonstrated that the unique arcade-like collagen fiber orientation in cartilage influences crack morphology and may help slow crack progression by ‘sealing’ cracks and maintaining fluid pressure during loading. Their finite element analysis highlighted the importance of collagen fibers in tissue mechanics and crack resistance (Moo et al., 2021). In the context of enzymatic degradation, Faisal et al. investigated the contribution of collagenase (MMP-1) and gelatinase (MMP-9) to fibrillar damage under mechanical loading. Their multiscale model showed that enzymatic activity, when combined with mechanical stress, can synergistically degrade collagen fibrils, illustrating the complex interaction between biochemical and mechanical factors in collagen damage (Faisal et al., 2023). These findings underscore that mechanical loading plays a significant role in collagen integrity in cartilage. Structural features such as fiber orientation, enzymatic activity, and joint biomechanics influence the extent and progression of collagen damage, which is central to both cartilage degeneration and repair processes.

## Strategies for cartilage repair and regeneration

Effective cartilage repair requires recreating the complex structure of the native ECM. Several strategies, including the use of biomaterials and tissue engineering, aim to restore the collagen network and enhance cartilage healing.

### Hydrogels mimicking the extracellular matrix

Recent advancements in hydrogel design have focused on recreating not only the biochemical but also the mechanical microenvironment of cartilage ECM. Native articular cartilage exhibits an equilibrium compressive (aggregate) modulus on the order of 0.7–0.8 MPa (Mow et al., 1980) and shear moduli of several hundred kilopascals, with pronounced stress relaxation and creep occurring over characteristic timescales of 10^2^–10^3^ s under physiological loading (Julkunen et al., 2008). By contrast, cartilage-mimetic hydrogels are typically engineered with compressive or storage moduli in the 10–1,000 kPa range: ECM-derived and cell-laden hydrogels commonly fall between 10 and 100 kPa, whereas highly crosslinked synthetic or double-network systems can reach approximately 200–1,200 kPa, still generally below native cartilage by at least one order of magnitude (Ngadimin et al., 2021). Dynamic covalent, supramolecular and physically crosslinked networks further allow tuning of stress-relaxation half-times from seconds to several hundred seconds, so that the relaxation profile of the hydrogel partially overlaps the stress-relaxation profile of native cartilage rather than only matching an instantaneous stiffness value. These tunable mechanical characteristics are crucial for replicating the complex viscoelastic behaviour of the native matrix, which exhibits both stiffness and toughness, and recent efforts aim to exploit this design space to guide chondrocyte phenotype maintenance and matrix deposition in three-dimensional scaffolds. Such tuning is enabled by hydrogels based on, for example, hyaluronic acid, gelatin, and photocrosslinkable or ionically crosslinkable alginate that can be formulated as injectable systems or bioinks, enabling precise control over scaffold architecture. Their injectability, printability and mechanical adjustability have driven significant progress, particularly through strategies that couple biochemical cues with quantitatively defined viscoelastic microenvironments.

### Chemically tunable hydrogels

Chemically tunable hydrogels, particularly those crosslinked through enzymatic or covalent reactions, provide a powerful platform to recreate cartilage-like mechanical microenvironments with quantitative control. In horseradish peroxidase (HRP)–mediated systems based on tyramine-functionalized polysaccharides such as hyaluronic acid–tyramine or dextran–tyramine, both gelation kinetics and stiffness can be modulated by tuning polymer concentration, degree of substitution and the HRP/H_2_O_2_ ratio. In such networks, the storage modulus is typically adjustable over at least one to two orders of magnitude, from a few kilopascals to several tens of kilopascals, and in hybrid or double-network formulations up to the 10^2^ kPa range, while gelation time can be shortened to ∼1–10 s or extended to several minutes depending on the crosslinking conditions. Similarly, covalently crosslinked PEG- and HA-based hydrogels, including double-network architectures, can be engineered with compressive or storage moduli on the order of 200–1,200 kPa, thereby approaching but still generally remaining below the ∼0.4–0.8 MPa equilibrium compressive modulus of healthy articular cartilage (Ngadimin et al., 2021). Together, these systems illustrate how polymer composition, functional group density and crosslinking chemistry jointly define a quantitative design space for cartilage-mimetic viscoelastic hydrogels.

### Collagen network engineering

In tissue engineering for articular cartilage repair, a major focus is the restoration of the anisotropic collagen network, which is crucial for maintaining the mechanical integrity and biological function of native cartilage. Recent advances have underscored the utility of scaffolds and biofabrication techniques in directing collagen fiber alignment during regeneration. A growing body of evidence supports the use of anisotropic scaffolds to promote organized collagen architecture. For example, bilayered extracellular matrix-derived scaffolds featuring anisotropic pore structures have been shown to guide collagen fiber orientation and facilitate hyaline-like cartilage repair in osteochondral defects (Browe et al., 2022). In parallel, biofabrication strategies have demonstrated significant efficacy. Incorporating chondroitinase ABC treatment with oriented fiber networks in polymer-based scaffolds has been reported to enhance collagen organization, highlighting the capacity of biofabrication to direct fibril alignment (Barcelo et al., 2023). Additionally, 3D printed scaffolds engineered with anisotropic guidance cues have been effective in promoting the maturation of cartilage tissue, particularly by supporting the formation of aligned type II collagen fibrils (Puiggali-Jou et al., 2024). Among emerging techniques, magnetic field-assisted alignment has shown promise. Specifically, the application of weak magnetic fields to align Fe_3_O_4_-coated silica nanorods within collagen matrices has enabled the fabrication of biomimetic scaffolds that replicate the native anisotropy of cartilage extracellular matrix (Wu et al., 2024; Patrawalla et al., 2024). Collectively, these innovative approaches—ranging from scaffold microarchitecture to magnetic alignment and 3D printing—demonstrate strong potential for reestablishing the directional collagen network essential for functional cartilage regeneration.

### MSC-based biologics as a complementary regenerative strategy

In addition to matrix-mimetic biomaterials, mesenchymal stem cells (MSCs) have become a major translational avenue for OA and cartilage repair (Bao and He, 2021). MSCs derived from bone marrow, adipose tissue, or perinatal tissues can be delivered via intra-articular injection, combined with scaffolds, or increasingly via cell-free MSC-derived extracellular vesicles (EVs). Across preclinical OA models, MSC-based interventions commonly demonstrate chondroprotective effects and improvements in joint homeostasis (Music et al., 2018; Jiang et al., 2024), while clinical evidence—summarized in recent randomized-trial meta-analyses—suggests that MSC injections can improve pain and function with an overall acceptable safety profile. However, structural endpoints (e.g., cartilage thickness on MRI) remain variable across studies, reflecting heterogeneity in OA stage, cell source, dose, and study design; placebo-related effects may also contribute to perceived benefit in some trials (Yin et al., 2025). Collectively, MSC strategies should be viewed as complementary to ECM- and mechanics-driven tissue engineering: biomaterials aim to reconstitute cartilage-like architecture and load-bearing function, whereas MSCs may primarily modulate the joint environment and support endogenous repair.

### Inflammation, macrophage polarization, and biomarker-informed therapeutic selection

OA is increasingly recognized as a whole-joint disease with a clinically meaningful inflammatory component, particularly synovitis driven by innate immune activation. Synovial macrophages can adopt pro-inflammatory (M1-like) or pro-resolving (M2-like) phenotypes; an elevated M1/M2 ratio is associated with synovial cytokine release (e.g., IL-1β, TNF-α, IL-6) and matrix catabolism mediated by enzymes such as MMPs and ADAMTS, whereas M2-like programs are linked to resolution signals (e.g., IL-10, TGF-β) and tissue repair (Oneto et al., 2025; Zhang et al., 2025). MSCs are well positioned within this framework because their paracrine factors/EVs can dampen inflammatory signaling and promote a shift toward anti-inflammatory macrophage responses, potentially reducing ongoing collagen/proteoglycan breakdown and facilitating reparative cascades in resident chondrocytes/progenitors. From a precision-medicine perspective, emerging biomarker domains may help stratify patients and guide strategy selection: inflammatory markers in serum/synovial fluid, cartilage matrix turnover markers, and imaging signatures of synovitis and cartilage integrity. While most soluble biomarkers are not yet standardized for routine clinical decision-making, integrating inflammatory phenotyping (e.g., synovitis-dominant OA) with matrix assessment could rationalize when immunomodulatory approaches (including MSC-based biologics) should be prioritized *versus* when primarily structural, ECM-mimetic scaffolds are more appropriate.

## Future perspectives

Future research in cartilage biology and repair must focus on refining our understanding of ECM–cell interactions at the microscale and how these translate to tissue-level mechanical behavior (Alonzo et al., 2020). Innovations in biofabrication, particularly those integrating spatial guidance for collagen fibril alignment, present promising avenues for engineering anisotropic scaffolds that more accurately mimic native cartilage architecture (Anbazhagan and Mahalingam, 2025). Additionally, the mechanobiological role of type III collagen in both tissue regeneration and OA progression warrants deeper investigation, as it may serve as a therapeutic target or biomarker (Yang et al., 2021; Wang et al., 2025). Computational modeling, including finite element simulations, should continue to be integrated with experimental data to predict tissue response under physiological and pathological loading conditions. Furthermore, the development of smart hydrogels with tunable stiffness, viscoelasticity, and biochemical functionality is critical for advancing scaffold designs that promote ECM remodeling and chondrocyte phenotypic stability. Combining these technologies with patient-specific modeling and bioprinting could significantly improve the efficacy and longevity of cartilage repair strategies.

## Conclusion

The structural complexity and functional specialization of articular cartilage are inherently tied to the dynamics of its ECM, particularly the organization and integrity of the collagen network. Pathological conditions such as OA underscore the importance of maintaining ECM homeostasis, as collagen degradation directly compromises mechanical functionality. Advances in biomaterials, including viscoelastic hydrogels and anisotropic scaffolds, have shown promising potential in replicating native ECM properties and promoting effective tissue regeneration. Moreover, understanding the differential roles of collagen types, particularly the reparative role of type III collagen, offers new directions for both diagnostics and therapeutics. Successful cartilage repair will ultimately depend on the integration of biomechanical, biochemical, and structural cues—requiring multidisciplinary approaches that bridge fundamental research with translational engineering.
